# Endoscopic management of submucosal tunnel abscess following upper esophagus submucosal tunneling endoscopic resection

**DOI:** 10.1055/a-2589-0880

**Published:** 2025-05-19

**Authors:** Shao-Bin Luo, Li Wang, Zu-Qiang Liu, Quan-Lin Li, Ping-Hong Zhou

**Affiliations:** 1 92323 Endoscopy Center and Endoscopy Research Institute, Zhongshan Hospital Fudan University, Shanghai, China; 2 Shanghai Collaborative Innovation Center of Endoscopy, Shanghai, China; 3 Endoscopy Center, Shanghai Geriatric Medical Center, Shanghai, China

A 24-year-old woman was admitted with a mass in the upper esophagus. Submucosal tunneling endoscopic resection (STER) was performed ( Video 1 ). The specimen was 3 cm × 2 cm, and the pathology was schwannoma.

Endoscopic management of submucosal tunnel abscess following upper esophagus submucosal tunneling endoscopic resection.Video 1

**Fig. 1:**
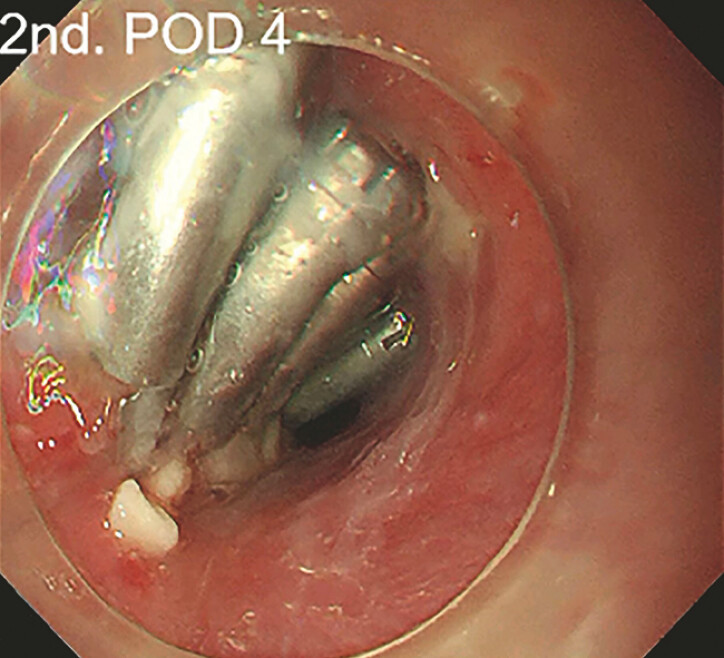
The second gastroscopy revealed the metal clip at the opening of the esophageal tunnel was in place.

**Fig. 2:**
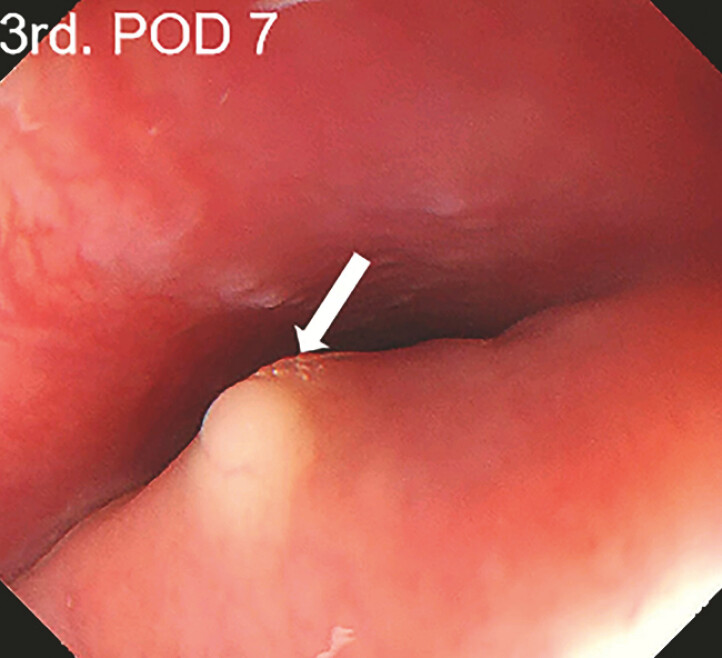
The third gastroscopy showed obvious mucosal swelling.

**Fig. 3:**
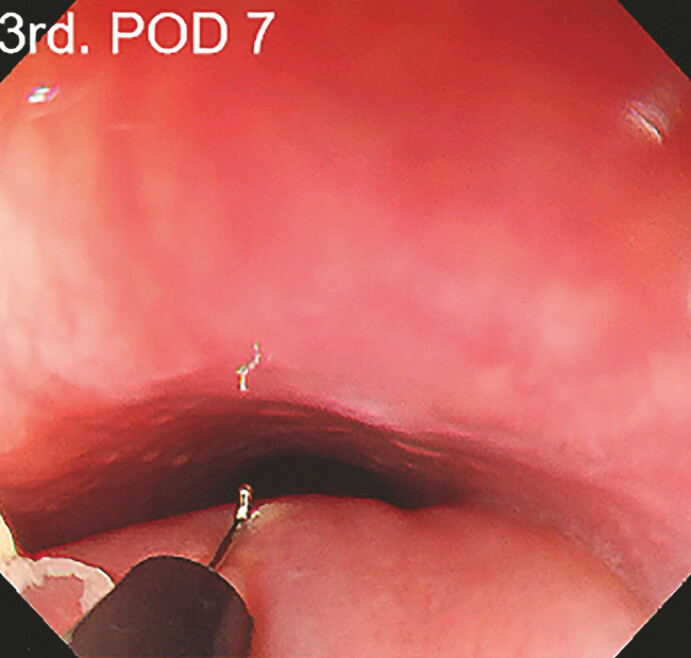
The mucosa was incised where the swelling is most obvious.

**Fig. 4:**
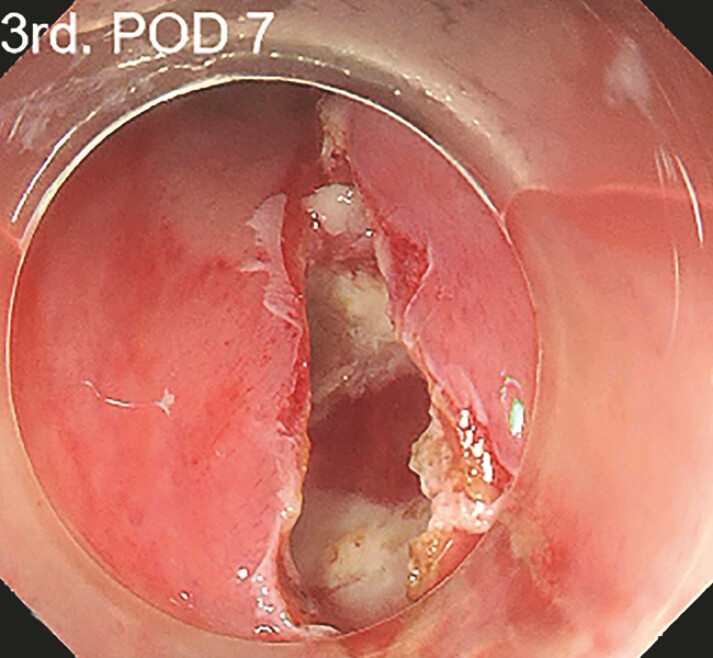
Cleared purulent secretion and adherence of purulent debris.

**Fig. 5:**
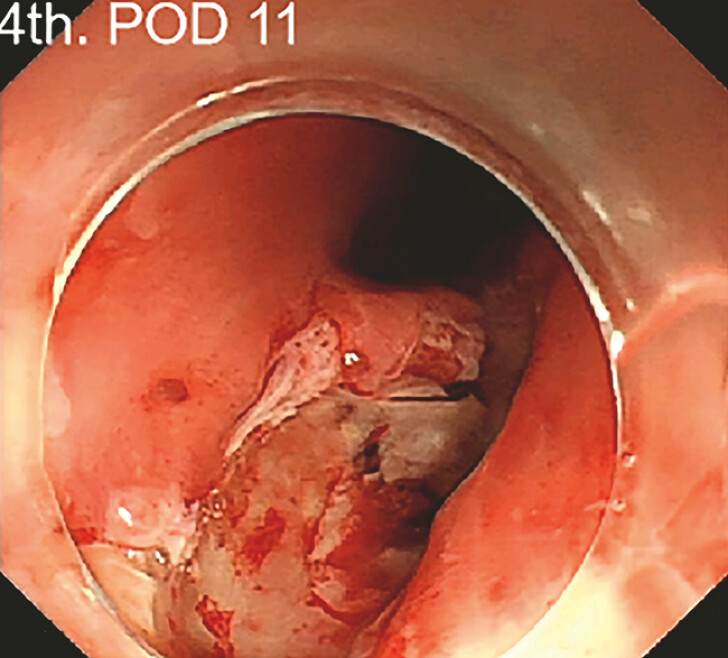
Follow-up endoscopy showed that the tunnel infection was controlled and fresh granulation tissue was seen in the residual cavity.

On postoperative day (POD) 3, the patient presented a fever with a maximum temperature of 38.5°C and chest tightness. No obvious inflammation, such as mediastinal and pleural effusion, was found by chest computed tomography. The second gastroscopy (POD 4) revealed that the metal clip at the opening of the tunnel was in place ( Fig. 1 ), and tunnel mucosal was normal. The patient still had a fever after 3 days of antibiotic treatment, and the third gastroscopy (POD 7) showed obvious mucosal swelling and a submucosal abscess at the middle of the tunnel ( Fig. 2 ). A large amount of pus can be seen gushing out after the incision at the most obviously swollen part of the mucosa ( Fig. 3 ). After extending the mucosal incision, the necrotic tissue was cleaned; the wound was not closed after being rinsed repeatedly, leaving it open for natural drainage ( Fig. 4 ), and the patient started drinking water after the procedure. The patients’ temperature quickly returned to normal, and the follow-up blood test examination on POD 8 showed a reduction in inflammatory markers. The fourth gastroscopy (POD 11) showed that the tunnel infection was controlled and granulation tissue had grown ( Fig. 5 ). The patient started drinking liquids 2 days later and was discharged the next day. Submucosal tunnel abscess is a rare major adverse event after STER, and the possibility of tunnel infection should be considered for fever, chest tightness, and sternal pain after STER without apparent cause. For the tunnel abscess, the swollen surface mucosa can be incised; after the pus and necrotic tissue in the tunnel are cleaned, the wound could be left open and naturally flushed and drained by drinking water.

Endoscopy_UCTN_Code_CPL_1AH_2AZ_3AZ

